# Generating evidence using electronic alerts during routine care: a fully automated randomized controlled trial of oral fluid restriction in acute heart failure (THIRST alert trial)

**DOI:** 10.1093/ehjdh/ztag098

**Published:** 2026-06-23

**Authors:** Yang Chen, Daniel Higgins, Yogini Jani, Nausheen Saleem, Kris Chafer, Ewan McFarlane, Timothy Roberts, Steve Harris, Bryan Williams, Matthew R Sydes, Folkert W Asselbergs, Anoop D Shah, R Thomas Lumbers

**Affiliations:** Institute of Health Informatics, Faculty of Population Health Sciences, University College London, Gower Street, London WC1E 6BT, UK; National Institute for Health and Care Research, University College London Hospitals Biomedical Research Centre, 250 Euston Road, London NW1 2PG, UK; Centre for Medicines Optimisation Research and Education, University College London Hospitals NHS Foundation Trust, 250 Euston Road, London NW1 2PG, UK; Centre for Medicines Optimisation Research and Education, University College London Hospitals NHS Foundation Trust, 250 Euston Road, London NW1 2PG, UK; Research Department of Practice and Policy, School of Pharmacy, University College London, London WC1E 6BT, UK; Centre for Medicines Optimisation Research and Education, University College London Hospitals NHS Foundation Trust, 250 Euston Road, London NW1 2PG, UK; National Institute for Health and Care Research, University College London Hospitals Biomedical Research Centre, 250 Euston Road, London NW1 2PG, UK; National Institute for Health and Care Research, University College London Hospitals Biomedical Research Centre, 250 Euston Road, London NW1 2PG, UK; Centre for Medicines Optimisation Research and Education, University College London Hospitals NHS Foundation Trust, 250 Euston Road, London NW1 2PG, UK; National Institute for Health and Care Research, University College London Hospitals Biomedical Research Centre, 250 Euston Road, London NW1 2PG, UK; Centre for Medicines Optimisation Research and Education, University College London Hospitals NHS Foundation Trust, 250 Euston Road, London NW1 2PG, UK; Institute of Health Informatics, Faculty of Population Health Sciences, University College London, Gower Street, London WC1E 6BT, UK; National Institute for Health and Care Research, University College London Hospitals Biomedical Research Centre, 250 Euston Road, London NW1 2PG, UK; National Institute for Health and Care Research, University College London Hospitals Biomedical Research Centre, 250 Euston Road, London NW1 2PG, UK; Innovative Clinical Trials Unit, Institute of Clinical Trials and Methodology, University College London, Gower Street, London WC1E 6BT, UK; Institute of Health Informatics, Faculty of Population Health Sciences, University College London, Gower Street, London WC1E 6BT, UK; National Institute for Health and Care Research, University College London Hospitals Biomedical Research Centre, 250 Euston Road, London NW1 2PG, UK; Department of Cardiology, Amsterdam Cardiovascular Sciences, Amsterdam University Medical Centre, University of Amsterdam, Meibergdreef 9, 1105 AZ Amsterdam, The Netherlands; Institute of Health Informatics, Faculty of Population Health Sciences, University College London, Gower Street, London WC1E 6BT, UK; National Institute for Health and Care Research, University College London Hospitals Biomedical Research Centre, 250 Euston Road, London NW1 2PG, UK; Institute of Health Informatics, Faculty of Population Health Sciences, University College London, Gower Street, London WC1E 6BT, UK; National Institute for Health and Care Research, University College London Hospitals Biomedical Research Centre, 250 Euston Road, London NW1 2PG, UK

**Keywords:** Pragmatic trials, Electronic health record, Clinical decision support system, Point-of-care randomization, Fluid restriction, Heart failure

## Abstract

**Aims:**

Many medical treatments lack robust evidence of safety and effectiveness from randomized controlled trials (RCTs). This is partly due to the cost and complexity of performing traditional RCTs and because randomization is not routinely embedded in clinical care. We aimed to evaluate the feasibility of conducting a pragmatic RCT fully integrated into the electronic health record (EHR) system to streamline patient identification, randomization, treatment allocation, and outcome assessment in patients admitted with acute heart failure.

**Methods and results:**

THIRST Alert was a single-centre parallel-group, open-label, feasibility RCT embedded in a hospital EHR system from May 3 to 1 November 2023. Adult patients who received more than one dose of intravenous furosemide within 48 h of admission were eligible. An interruptive alert was triggered when physicians accessed the medication order chart of eligible patients, inviting them to enrol the patient in the study. Enrolled patients were randomized to either ‘oral fluid restriction of 1 L per day’ or ‘no fluid restriction’. The co-primary feasibility outcomes were the total number of patients recruited and the documented difference in oral fluid intake within 48 h of randomization. Twenty-three patients (16%) were enrolled from 145 eligible patient admissions; there were no repeat admissions among enrolled patients. A total of 1191 enrolment alerts were triggered, reaching 216 individual clinicians. 22/23 trial participants (96%) had a diagnosis of heart failure. No significant difference in oral fluid intake was observed between the treatment groups (median difference 518 mL; 95% confidence interval: −235 to 1270; *P* = 0.18). Documented oral intake was numerically higher in the oral fluid restriction group (1168 mL; interquartile range [IQR] 932–1620 mL) compared to the group without restriction (650 mL; IQR 75–1102 mL), which may reflect documentation bias rather than a true increase in oral fluid intake or a lack of intervention efficacy.

**Conclusion:**

This study demonstrates the feasibility of a pragmatic RCT fully integrated within a hospital EHR system in acute care. Although alert-to-enrolment rate was modest, the overall recruitment rate was comparable to conventional acute care RCTs, highlighting the potential of EHR-embedded trials to efficiently address evidence gaps in the management of conditions such as acute heart failure.

**ClinicalTrials.gov:**

NCT05869656.

## Graphical Abstract

**Graphical Abstract:**
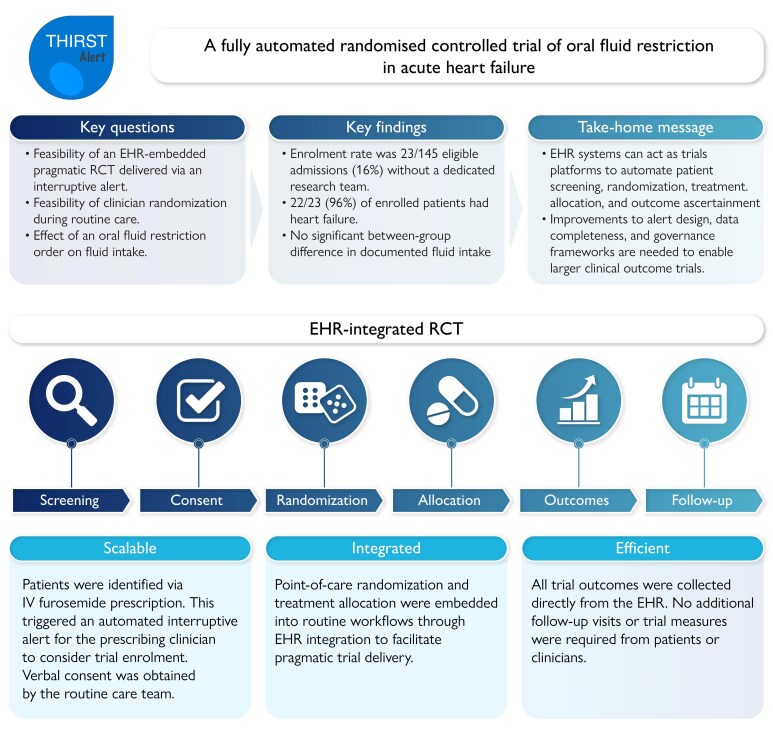

## Introduction

Randomized controlled trials (RCTs) are complex and expensive to conduct in healthcare settings because they require a parallel infrastructure for research, including dedicated personnel, data collection systems, and processes that operate alongside routine clinical care.^1^ Consequently, there is uncertainty regarding the safety and effectiveness of many commonly used treatments, particularly in acute, unplanned care.^2^ Pragmatic trial designs that fully integrate RCT procedures into the electronic health record (EHR) system, and harness computerized decision support systems (CDSS) as interventions, may represent an efficient solution to generate evidence in such settings; however, the feasibility of this approach has not yet been established.^3^ While a growing number of CDSS are deployed within EHR systems, they typically focus on applying existing evidence rather than supporting the recruitment to EHR-embedded pragmatic RCTs designed to generate new evidence.^4,5^

Among the many evidence gaps in acute cardiovascular care, the use of oral fluid restriction in patients treated for acute heart failure (HF) is a prominent example highlighted in professional guidelines.^6,7^ Acute HF is the leading cause of unplanned hospitalization in patients over 65 years of age and is defined by fluid overload.^8,9^ While intravenous loop diuretics are the standard of care for patients with fluid overload due to HF or other causes, including liver and renal disease, restriction of oral fluid intake is often implemented as an adjunctive treatment.^10,11^ The safety and effectiveness of this intervention, however, have not been established. Three small RCTs of oral fluid restriction in acute HF yielded inconclusive results: two suggested no clinical benefit, and all reported increased thirst with fluid restriction.^12–14^

Here, we report the findings of a pragmatic RCT fully embedded within an EHR system, designed to assess the feasibility of this novel approach to clinical trials delivery.

The THIRST Alert trial aimed to (i) assess the feasibility of using an automated interruptive alert to invite routine care clinicians to enrol patients with fluid overload into an RCT of oral fluid restriction, and (ii) examine the effect of an oral fluid restriction order on documented oral fluid intake. Oral fluid intake was selected as a co-primary outcome based on its clinical relevance and availability, and to evaluate its suitability as a potential measure of intervention adherence for subsequent outcome-driven studies. Although measurement quality in routine care is often poor, we hypothesized that fluid intake documentation may improve in this pragmatic RCT setting due to a Hawthorne effect on the clinical care team.^15,16^ The pragmatic trial design would additionally reduce the Hawthorne effect that patients would experience owing to a streamlined set of trial procedures. To our knowledge, THIRST Alert represents the first low-friction, fully automated pragmatic RCT seamlessly integrated into the EHR and routine clinical workflow. We sought to determine methodological feasibility and to use the results to inform the design of larger trials powered for clinical outcomes.

## Methods

### Study design

THIRST (Randomized Controlled **T**rial within the electronic **H**ealth record of an **I**nterruptive alert displaying a fluid **R**estriction **S**uggestion in patients with the treatable **T**rait of congestion) Alert was a single-centre parallel group, open-label, randomized controlled feasibility trial conducted at University College Hospital (UCLH), a digitally mature hospital.^17^ The study protocol was approved by the London Riverside Research Ethics Committee (Ref: 22/LO/0889) and the study was registered with ClinicalTrials.gov (NCT05869656). The full study protocol has been reported previously.^18^ The study was conducted and reported in accordance with CONSORT and the relevant extensions (CONSORT pilot and feasibility, CONSORT-Routine) and the TIDieR checklist.^19–21^

**Figure 1:**
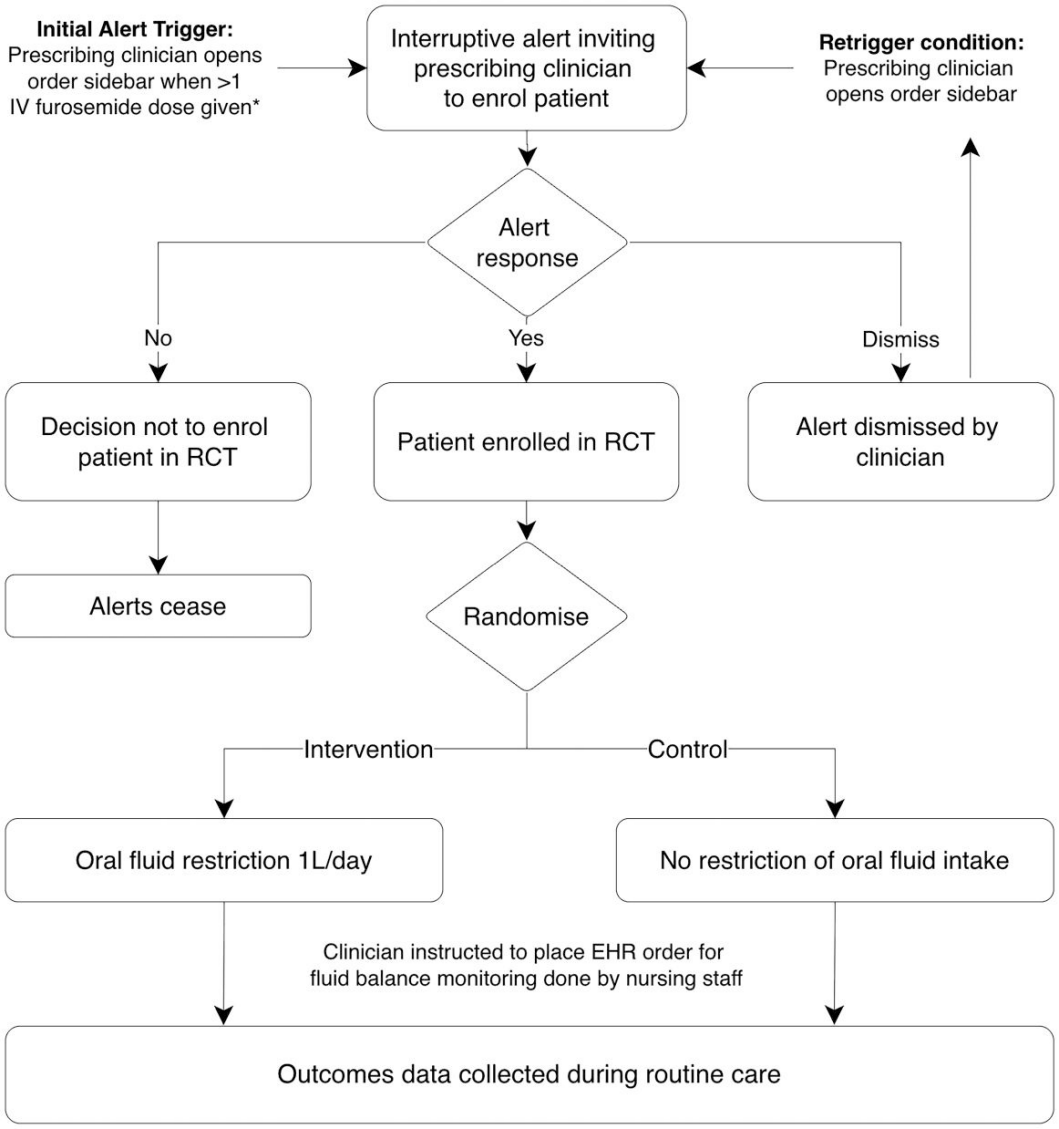
Programme theory for THIRST alert trial. *Patient identification and presentation of the alert to the prescribing clinician required presence of a second IV furosemide order within 48 h of admission. The alert design allowed users to select ‘Yes’, ‘No’ or ‘Dismiss’ when invited to enrol the patient into the trial. Yes/No replies silenced the alert. ‘Dismiss’ allowed the alert to be presented again, if <48 h had elapsed. © 2024 Epic Systems Corporation.

The trial was designed to pilot a novel approach that fully integrates trial procedures within the EHR and is delivered in routine care, without a dedicated research team. Patient screening, consent, randomization, treatment allocation, and outcome ascertainment were all conducted through the EHR and routine care processes (*Figure 1*). The trial intervention and programme theory were co-designed with a multidisciplinary team including prescribing clinicians (physicians), nurses, health informaticians, and patient representatives. EHR-based patient identification, point-of-care randomization, and alert triggering were tested using a staged approach, including a silent mode of operation where the alert was ‘on’ but not visible to staff. A patient advisor (KC) reviewed all patient-facing materials.^22^

### Participants

Adults ≥18 years prescribed more than one dose of intravenous (IV) furosemide within 48 h of hospital admission were eligible. The use of prescription data as a surrogate for fluid overload and acute HF was pragmatic, relying only on complete prescription data and a simple identification rule that does not depend on the accurate and timely coding of structured diagnostic data in routine care. Once patients satisfied this rule, an interruptive enrolment alert was triggered whenever a clinician with prescribing rights opened the medication orders section of the record.

### Enrolment workflow

The interruptive enrolment alert stated: ‘Your patient is eligible for the THIRST Alert pragmatic clinical trial. They would be randomised to either oral fluid restriction (1 L per day) or to continue with free fluids. Do you agree with randomisation?’ Clinicians could respond by selecting Yes, No, or Dismiss, exercising their clinical judgement in deciding whether to enrol the patient. If the clinician selected Dismiss, the alert would continue to be retriggered within a 48 h window after admission; selecting No would cease further alerts for that patient admission. If the clinician selected Yes to enrol the patient, randomization was triggered. The allocated treatment was presented to the clinician in a separate alert, which included instructions to inform the patient about the study and obtain verbal consent. A standardized one-page participant information sheet (PIS) for patients was also provided in the alert via a hyperlink. Patients were informed that they could withdraw from the study at any stage during their admission.

### Randomization

Following the clinician’s acceptance of enrolment, participants were randomized using a 1:1 randomization method to either oral fluid restriction or no restriction, implemented using a purpose-built Epic randomization tool and internal random number rule. No block randomization or additional covariate balancing was undertaken. Given the focus on methodological feasibility, we used simple randomization to enable a low-complexity, fully automated EHR-based implementation. Any imbalance in group sizes or baseline characteristics would inform the design of a future effectiveness trial. As clinicians were unblinded, restricted randomization methods were avoided to minimize allocation predictability, which might bias the enrolment decision. Treatment allocation was presented to prescribing clinicians in the form of a second alert with the same trigger condition. For patients randomized to the intervention group, the clinician received a recommendation for a target oral fluid restriction of 1 L per day; for patients allocated to the control group, the care team received a recommendation for no restriction in oral fluid intake. For both randomized groups, the alert provided additional instructions to the clinician to (1) complete a nursing order for fluid balance monitoring; (2) document the treatment allocation in the clinical notes; (3) inform the patient and the nursing team; and (4) provide the patient with a PIS. The nursing team received a notification of the treatment allocation and instruction to conduct regular fluid balance monitoring within their routine workflow. The study was open label.

### Intervention

The intervention consisted of an interruptive alert delivered through the EHR, providing an enrolment invitation and a treatment recommendation for patients who were enrolled. The first alert invited clinicians to enrol eligible patients into the study. After enrolment, a second alert communicated the randomized treatment allocation: either oral fluid restriction of 1000 mL per day or no oral fluid restriction. The target restriction of 1000 mL per day of oral fluid was selected to balance safety with the potential for a clinically meaningful difference, informed by prior studies.

### Outcomes

The co-primary feasibility outcomes were (i) the total number of patients recruited during the trial recruitment period and (ii) the documented difference in oral fluid intake between the two groups within 48 h of randomization. Fluid intake was documented by nursing colleagues on the EHR as part of their routine clinical work. Documentation was performed by nursing staff at times when clinical service demands allowed. This was variable and event-driven rather than recorded on a regular schedule.

Secondary trial endpoints included process measures, such as adherence to alert recommendations, and clinical measures, such as weight change between groups, frequency of renal monitoring, and diuretic dosing. Adherence to the recommended treatment allocation was assessed by team implementation, defined as physician acknowledgement (completion of a nursing order for fluid balance monitoring), nursing acknowledgement (acknowledgement of the fluid balance monitoring order), or both. An ordinal scale was used to rank the level of adherence to the allocated treatment, from 0, indicating no evidence of adherence, to 6, indicating acknowledgement by both treating clinician and nursing staff within 12 h of randomization (see Supplementary material online, *Table S1*).

### Statistical analysis

No formal sample size calculation was performed because the co-primary outcomes assessed feasibility.^23^ The study was not powered to detect differences in clinical endpoints. Pre-specified feasibility success criteria were recruitment of more than 20 participants during the study period and a between-group difference of 250 mL, based on site recruitment capacity and oral intake differences observed in previously reported trials. Progression to a full outcomes trial was contingent on meeting these criteria.

Baseline characteristics of study participants were summarized using percentages, medians, and interquartile ranges (IQRs). Patients enrolled in the trial were compared to the pool of eligible patients who were not enrolled, using the Kruskal–Wallis test or χ^2^ test for proportions. For randomized patients, the outcomes between treatment groups were compared using *t*-test or Mann–Whitney U-test for continuous outcomes. Between-group effects were summarized as the difference in medians (oral fluid restriction minus no oral fluid restriction). This approach was applied to oral intake (mL) and to the count of oral-intake documentation entries. Using a nonparametric bootstrap, 95% confidence intervals (95% CI) for median differences were obtained. Comparisons in the number of data entries for oral intake according to trial participation, treatment allocation or implementation status between relevant groups was made using the Mann–Whitney U-test. Alerts with a definitive response were compared to alerts that were dismissed, in terms of staff professional role and occurrence during night shift periods, using the χ^2^ test for proportions. No adjustment or imputation was used for missing data. A *P* value <0.05 was considered statistically significant, with Bonferroni adjustment for multiple comparisons. The analysis was conducted on an intention-to-treat basis. All analyses were performed using R software version 4.1.3 (R Foundation for Statistical Computing).

### Data management

All trial data, including alert meta-data and outcomes, were extracted from the EHR. Most clinical variables were extracted in the Observational Medical Outcomes Partnership Common Data Model format.^24^ Primary and secondary diagnoses for enrolled patients were determined using International Classification of Disease (ICD)-10 codes from administrative billing data of admitted care episodes and SNOMED CT terms from structured problem lists. ICD-10 and SNOMED CT code lists used to define study co-variates are provided in the supplement. No data linkage was required. Study data were analysed in a secure research environment within UCLH NHS Foundation Trust. Manual adjudication of outcomes and co-variates was undertaken in enrolled patients.

### Protocol amendment

During trial monitoring, clinicians received a greater number of alerts than expected. The trial management group adjusted the triggering condition after 20 days to reduce the possibility of alert fatigue.^25^ The triggering condition ‘Open Patient Chart’ was removed, leaving ‘Open Orders Sidebar’ as the sole alert trigger (see Supplementary material online, method).

## Results

**Figure 2:**
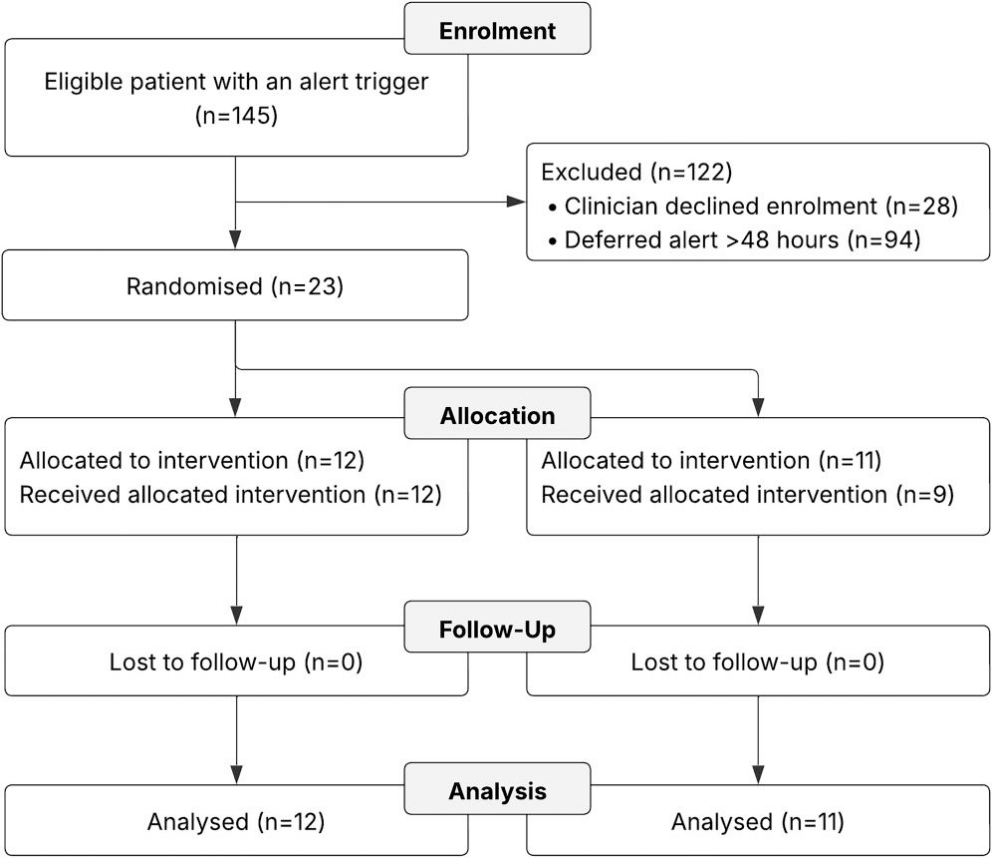
CONSORT flow diagram.

**Figure 3:**
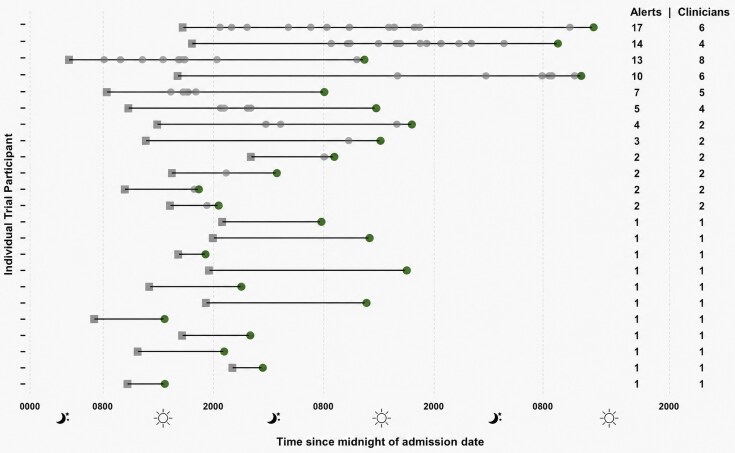
Clinical responses to enrolment alert recorded on EHR in hours following decision to admit patient. Square = Decision to admit, Circle = Enrolment alerts presented to a prescribing clinician. Grey colour represents a ‘dismiss’ response and green colour ‘yes’ to enrolment.

Between 3 May 2023 and 1 November 2023, of 145 eligible patient admissions, 23 patients (16%) were enrolled to the trial by routine care clinicians; there were no repeat admissions among enrolled patients (*Figure 2*). In total, 1191 enrolment alerts were triggered and presented to 216 individual prescribing clinicians. Following the protocol amendment, the median number of alerts per patient decreased from 12 (IQR 6–22) to 6 (IQR 2–9) (see Supplementary material online, Methods; Supplementary material online, *Figure S1*). Among patients who were enrolled, the median number of alerts prior to enrolment was 2 (IQR 1–4.5), and the median time between decision to admit and enrolment was 12 h (IQR 8–26) (*Figure 3*). On days when physicians received a THIRST trial enrolment alert, they were also exposed to a median of 28 unrelated EHR alerts during routine patient care (IQR 16–42). There were no significant differences in proportions of clinician professional grade or out-of-hour alerts between those providing a definitive response and those who dismissed the alert (see Supplementary material online, *Table S2*). National resident doctor strike action affected 21 of the 153 days during the study recruitment period, with only one patient recruited during those days.

Among eligible patients, baseline characteristics were not associated with the physician's decision to enrol, decline enrolment, or defer, in response to the THIRST Alert invitation, Bonferroni-adjusted *P* > 0.05 (*Table 1*). Enrolled patients had a median age of 79 (IQR 74–87); 61% were women and 30% were of non-White ethnicity. Baseline characteristics were similar between the randomized groups. The oral fluid restriction group, however, included fewer women (*n* = 4 vs. *n* = 10) and had higher median NT-proBNP levels (3116 ng/L, IQR 1139–5322) compared to the no restriction group (1736 ng/L, QR 646–4420) (see Supplementary material online, *Table S3*). Of the 23 patients enrolled, 22 (96%) had a confirmed diagnosis of HF. 19/23 (83%) had a documented diagnosis in structured data, with HF coded as the primary cause for admission in 14 patients (61%). Of the remaining four patients, a HF diagnosis was confirmed in three after case note review. One patient had fluid overload from a condition other than HF (see Supplementary material online, *Table S4*).^26^

**Table 1:** Characteristics of patients (1) enrolled (2) declined enrolment (3) deferred = not enrolled within time window.

	Enrolled	Not Enrolled	
Clinician action	Enrolled (*n* = 23)	Declined (*n* = 28)	Deferred >48 h (*n* = 94)
Age	79 (74–87)	76 (61–90)	79 (69–86)
Female	14 (61)	10 (36)	40 (43)
Asian	4 (17)	3 (11)	11 (12)
Black	1 (4)	4 (8)	8 (9)
Other	2 (9)	1 (4)	16 (17)
White	12 (52)	14 (50)	43 (46)
Ethnicity not stated	4 (17)	6 (21)	16 (17)
HF as primary cause	14 (61)	13 (46)	40 (43)
HF as contributory cause	5 (22)	9 (32)	39 (42)
Non-HF diagnosis	4 (17)	6 (21)	15 (16)
Chronic kidney disease (CKD)	7 (30)	8 (29)	26 (28)
CKD stage 2–3	3 (13)	2 (7)	8 (9)
CKD stage 4–5	1 (4)	3 (11)	4 (4)
CKD unspecified	3 (13)	3 (11)	14 (15)
Acute kidney injury	4 (17)	12 (43)	23 (25)
Chronic liver disease	1 (4)	1 (4)	2 (2)
Chronic pulmonary disorders	8 (35)	14 (50)	23 (25)
Atrial arrhythmias	6 (26)	12 (43)	38 (40)
Coronary artery disease	8 (35)	3 (11)	35 (37)
Diabetes	7 (30)	11 (39)	35 (37)
Hypertension	13 (57)	12 (43)	51 (54)
BMI^a^	29.3 (25.5–30.6)	26.3 (21.6–31.2)	26.0 (22.5–31.0)
Systolic BP, mmHg	142 (117–152)	134 (119–176)	138 (120–164)
Diastolic BP, mmHg	74 (63–83)	69 (64–86)	74 (67–86)
Heart rate, beats/min	84 (73–93)	86 (73–91)	86 (70–108)
Creatinine, umol/L^a^	94 (78–133)	93 (74–133)	93 (70–126)
eGFR, mL/min/1.73 m^a^	54 (36–74)	54 (42–76)	60 (41–79)
NT-pro BNP, ng/L^a^	1795 (950–4915)	2507 (896–10596)	3504 (1362–7806)
Left ventricular ejection fraction (LVEF)^b^	47 (31–59)	60 (38–60)	55 (30–60)

Data are median (IQR) or *n* (%).

^a^BMI data availability: 22/23 enrolled, 25/28 not enrolled, 83/94 deferred; Creatinine and eGFR data availability: 1/94 timed out; NT-proBNP data availability: 22/23 enrolled, 23/28 not enrolled, 75/94 deferred.

^b^Numeric LVEF data was available (10/23 enrolled, 9/28 not enrolled, 31/94 timed out).

BMI, body mass index; CKD, chronic kidney disease; eGFR, estimated glomerular filtration rate; LVEF, left ventricular ejection fraction; HF, heart failure.

Adherence to the randomized treatment allocation was assessed based on the presence or absence of physician EHR orders and their acknowledgment by nursing staff. Within 48 h of enrolment, 18/23 (78%) of patients had physician acknowledgement, of whom 12/23 (52%) also had subsequent nursing acknowledgement (see Supplementary material online, *Table S5*, Supplementary material online, *Figure S2*). EHR evidence of treatment implementation (either physician, nursing or both) was not associated with the number of oral fluid intake data entries recorded in 48 h (median 5 [IQR 1.5–8.5] vs. 8 [IQR 7–8]); median difference −3 (95% CI: −6 to 4) (see Supplementary material online, *Figure S3*).

Documented oral fluid intake during the 48 h study period following randomization was similar between the treatment groups (median difference 518 mL; 95% CI: −235 to 1270; *P* = 0.18) The median intake was numerically higher in the fluid restriction arm (1168 mL; IQR 932–1620) vs. the no restriction arm (650 mL; IQR 75–1102). This likely reflects documentation bias rather than a true increase in oral fluid intake or non-adherence to the treatment protocol. The median number of oral fluid intake data entries was 4 (IQR 1–7) for the no fluid restriction group vs. and 7 (IQR 4–9) for the fluid restriction group (median difference 3 entries; CI: −2 to 7). In both groups, the documented daily oral fluid intake was less than 1000 mL (see Supplementary material online, *Table S6*). Enrolled patients overall had significantly more fluid intake entries than non-enrolled patients (6 vs. 2), median difference 4 entries (95% CI 1–6; *P* = 0.01), suggesting that trial participation increased documentation frequency. In the subgroup of patients with HF coded as the primary cause for admission (14/23 patients), median oral fluid intake was 1250 mL (IQR 1068–1640) in the restriction group vs. 955 mL (IQR 510–1580) in the no restriction group (median difference 295 mL (95% CI −955 to +950) (see Supplementary material online, *Figure S4*).

### Secondary outcomes

Secondary outcome results are presented in Supplementary material online, *Tables S7*–S8. Weight change was available in 11/23 enrolled patients (48%). There was negative weight change in 5/7 of the oral fluid restriction group and 3/4 patients in the no oral fluid restriction group. The sample size was insufficient to draw meaningful conclusions. Change in creatinine was available in 14/23 enrolled patients (61%). No structured data for patient-reported outcome measures of thirst were recorded during the study. No patients withdrew from the trial, and no serious adverse events were attributed to the study intervention.^27^

## Discussion

Our findings demonstrate the feasibility of conducting an automated, EHR-embedded pragmatic RCT in patients with acute HF within routine clinical care. Repeated IV furosemide prescription provided a reliable trigger for patient identification, avoiding reliance on diagnostic coding, which is often incomplete.^28^ An interruptive alert to the prescribing clinician served as a practical recruitment mechanism. Verbal opt-out consent, delivered without a dedicated research team, was acceptable to patients and clinicians and raised no safety concerns. Outcomes data were, however, incomplete highlighting the importance of standardized routine care pathways that reliably capture relevant outcomes.

Interruptive EHR alerts inviting routine care clinicians to enrol eligible patients were an effective recruitment mechanism: a median of two alerts was triggered prior to enrolment, and most patients were enrolled within 24 h of admission. The alert-to-enrolment rate of 16% was modest. Comparisons with other studies are difficult because screening-to-randomization rates in acute care RCTs are rarely reported. However, a 2015 review of 26 studies reported a median yield of 47% (range 2%–98%), with lower rates for non-pharmaceutical interventions and hospital-based trials; lower recruitment rates were also reported for time-sensitive acute care studies.^29,30^ We did not identify any specific patient characteristics that were significantly associated with the clinician decision to accept, decline, or defer enrolment; however, power was limited. Interruptive alerts rely on behavioural change to achieve their intended effect, and the exposure of clinicians across different specialties and seniority levels in our study demonstrates that even a simple digital alert constitutes a complex intervention.^31^ Compared to other studies of EHR alerts, we focused on downstream clinical effects including fluid intake as a co-primary outcome, rather than solely examining process measures such as the percentage of alerts responded to.^32^

The difference in oral fluid intake between the groups did not reach significance. Unexpectedly, the documented intake was numerically higher in the fluid restriction arm, likely reflecting documentation bias (a form of measurement artefact influenced by a Hawthorne effect) rather than non-adherence. Enrolment into the trial was associated with higher rates of oral fluid intake documentation compared with the eligible population who were not enrolled, with a trend towards increased measurement frequency and documentation in the active treatment arm. Despite this, documentation of fluid intake was incomplete; for example, five patients had a documented oral fluid intake of less than 500 mL in 48 h. These observations are consistent with documentation bias, a common limitation of studies of behavioural interventions.^16^

The need to improve the efficiency and cost-effectiveness of clinical trials^33^ has driven increasing use of EHR data in trials, typically for participant identification^34^ or outcome ascertainment.^35^ Most RCTs using EHR have continued to depend on conventional study teams to recruit and consent participants. Fully EHR-embedded trials reduce the reliance on a dedicated research team and offer advantages in acute care settings, where timely recruitment is challenging. Recent examples include RCTs of antibiotic regimes for *Pseudomonas* and of magnesium supplementation in critical care.^36,37^ To our knowledge, the THIRST Alert trial is the first example of a fully EHR-embedded pragmatic RCT in acute cardiovascular care.

### Limitations

There are several limitations to our feasibility study.

First, although there is no reported benchmark for alert-to-enrolment rates, the yield was modest in our study. To minimize disruption to clinical care, we did not include a qualitative evaluation of the reasons for clinicians’ decisions not to enrol; this may have provided additional information to better understand the drivers of non-enrolment.^37^

Second, data completeness for certain study covariates and outcomes was limited due to the constraints of data extraction from unstructured fields. Despite design features to improve data capture, we were constrained in our ability to add new EHR functionality or alter clinical workflows. Missing or incomplete data were notable for the secondary outcomes such as weight change and structured documentation of thirst. Echocardiographic data were available for most patients (22/23 [96%] enrolled; 107/122 [88%] unenrolled); however, structured numerical left ventricular ejection fraction values were available in fewer patients (10/23 [43%] enrolled; 40/122 [33%] unenrolled). Data completeness is also limited by routine documentation practices, which vary with clinical factors (e.g. patient acuity) and non-clinical factors (e.g. staffing and time of day). Balancing additional trial-related procedures with routine care to improve data quality remains challenging and highlights the importance of feasibility studies. Although we did not observe evidence of crossover between arms, behavioural interventions delivered through routine care systems are inherently susceptible to contamination and documentation bias. Intervention implementation and outcome ascertainment depended on routine care documentation, which is influenced by the care context and may vary systematically by treatment group. Documentation of fluid intake, for example, may have been influenced by allocation to the active treatment arm.^38^

Third, while facilitating the delivery of a feasibility trial, our decision to randomize at the patient level rather than the provider or cluster level increased the possibility of treatment contamination across groups.^39^ We could not formally quantify the effect of this, given that the treatment was behavioural. Cluster-level randomization (e.g. by ward or hospital) or stepped-wedge cluster designs could reduce contamination by enabling whole-team adoption of one or another treatment; both approaches should be considered in the design of larger studies.^40^

Fourth, we noted that our initial alert trigger was too sensitive in the first three weeks of the trial recruitment window. This required the return of the alert to a ‘shadow period’ for one month to allow the triggering conditions to be modified. The increased sensitivity of the initial alert may have impacted on subsequent clinicians’ receptiveness to engaging with the alert. The impact of clinician-specific selection bias was not assessed. However, the modest recruitment rate and large number of clinicians exposed to the initial alert emphasizes that although alert fatigue may be a significant concern, the total number of trial alerts that each clinician experiences is a fraction of all other alerts within the EHR. Resident doctor strikes affected 13.7% of the trial recruitment window. During strike days, emergency cover was provided by senior doctors, which may have influenced recruitment.

### Implications

Although our findings are based on a specific EHR vendor, the trial procedures should be replicable across other systems; however, the potential influence of different interface design on alert acknowledgement warrants further study.^41,42^ We have shown that EHRs can function as full RCT platforms rather than solely to aid patient identification or for the collection of clinical outcomes data.^43–45^ EHR alerts can ‘nudge’ clinicians to recruit patients into pragmatic, low-risk trials, with outcomes ascertained directly from the care record.^46^ This approach represents a scalable approach to generate randomized evidence for treatments of uncertain efficacy and/or safety, extending beyond their use as guidelines adherence reminders that have been most widely studied.^2,47,48^

For future studies in which outcomes depend on manual documentation, workflows should be designed to support scheduled measurements rather than event-driven reporting. Clinical outcomes, such as readmission and mortality, will be critical for larger studies and will rely on national data linkage. Finally, variation in governance and consent frameworks^49,50^ is likely to be a major determinant of feasibility across different jurisdictions.^51–53^

## Conclusion

THIRST Alert demonstrates the feasibility of conducting an automated, EHR-embedded pragmatic RCT in acute HF without direct research team involvement. The alert-to-enrolment rate was modest but consistent with conventional trials in acute care settings. Limitations in data completeness highlight opportunities to refine the design of larger studies. Overall, these findings demonstrate the feasibility of scalable EHR-embedded trial infrastructure, offering an opportunity to address persistent evidence gaps in acute cardiovascular care.

## Supplementary Material

ztag098_Supplementary_Data

## Data Availability

The data underlying this work are available in the main article and online Supplementary material. Additional data are available upon reasonable request to the first author.
