# Supplementary material for: Generating evidence using electronic alerts during routine care: a fully automated randomized controlled trial of oral fluid restriction in acute heart failure (THIRST alert trial)
Source: Eur Heart J Digit Health. 2026 Jun 23;7(6):ztag098. doi: 10.1093/ehjdh/ztag098 (PMC13335805; doi:10.1093/ehjdh/ztag098)
Supplement: ztag098_Supplementary_Data [file ztag098_supplementary_data.zip › 2026-03-26_THIRST paper supplement v2.docx]

# Supplementary Note

Supplementary Tables

Supplementary Figures

Supplementary Methods

## Supplementary Tables

| **Adherence** | **Example/ notes** |
| --- | --- |
| 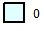 | No EHR footprint of a follow up action after randomised allocation |
| 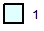 | Evidence of Physician Acknowledgement following randomised allocation:  |
| 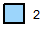 | Evidence of Physician Acknowledgement following randomised allocation ***within 48 hours*** |
| 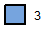 | Evidence of Physician Acknowledgement following randomised allocation ***within 48 hours***    **AND**  Evidence of Nursing Acknowledgement within 48h   |
| 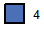 | Evidence of Physician Acknowledgement following randomised allocation ***within 48 hours*** +  Evidence of Nursing Acknowledgement within 36h |
| 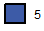 | Evidence of Physician Acknowledgement following randomised allocation ***within 48 hours*** +  Evidence of Nursing Acknowledgement within 24h |
| 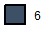 | Evidence of Physician Acknowledgement following randomised allocation ***within 48 hours*** +  Evidence of Nursing Acknowledgement within 12h |

**Table S1: Degree of documented acknowledgement of treatment allocation.** An ordinal scale was used to rank the strength of adherence to the allocated treatment group, 0 were admissions with no documented evidence of adherence to the allocated treatment and 6 were admissions with physician and nursing acknowledgement of allocation treatment, within 12 hours of randomisation. The same colour gradient is used in Supplemental Figures 2 & 3. © 2024 Epic Systems Corporation

|  | **Admissions with**  **patient enrolment** | **Admissions without patient enrolment** |
| --- | --- | --- |
| Number of enrolment alerts | 112 | 1079 |
| Number of alerts acknowledged with definitive action | 23 | 36* |
| Alerts per admission | 2 (1-6) | 7.5 (4-12) |
| Number of clinicians alerted per admission | 2 (1-4) | 4 (2-5) |
| Unique number of clinicians exposed | 46 | 207 |
| Foundation year doctor | 16 (35) | 44 (21) |
| Specialty registrar | 24 (52) | 133 (64) |
| Consultant | 6 (13) | 30 (15) |

**Table S2a. Alert and user characteristics.** Data are median (IQR) or n (%). Clinicians exposed to the alert who clicked dismiss during the study period reset the alert for repeat presentation until Yes/ No selected or 48 hours had elapsed. *13 duplicates during first 3 week period

|  | **Enrol alerts (n=23)** | **Decline alerts (n=23)** | **Alert with dismiss (n=1132)** | ***P value*** |
| --- | --- | --- | --- | --- |
| Foundation year doctor | 4 (17) | 7 (30) | 291 (26) | 0.58 |
| Specialty registrar | 16 (70) | 15 (65) | 748 (66) | 0.94 |
| Consultant | 3 (13) | 1 (4) | 92 (8) | 0.55 |
| In hours^+^ | 10 (43) | 11 (48) | 632 (56) | 0.38 |

**Table S2b. User characteristics and time of enrolment alert**. Data are n (%) ^+^from 9am to 6pm inclusive

|  | **Fluid Restriction (n=12)** | **No Fluid Restriction (n=11)** |
| --- | --- | --- |
| Age | 81 (71-87) | 77 (75-86) |
| Female | 4 (33) | 10 (91) |
| Asian | 4 (33) | 0 (0) |
| Black | 0 (0) | 1 (9) |
| Other | 0 (0) | 1 (9) |
| White | 4 (33) | 8 (73) |
| Ethnicity not stated | 4 (33) | 0 (0) |
| HF as primary cause | 7 (58) | 7 (64) |
| HF as contributory cause | 3 (25) | 2 (18) |
| Non-HF cause | 2 (17) | 2 (18) |
| Chronic kidney disease (CKD) | 4 (33) | 3 (27) |
| Chronic liver disease | 1 (8) | 0 (0) |
| Chronic pulmonary disorders | 4 (33) | 4 (36) |
| Atrial arrhythmias | 4 (33) | 2 (18) |
| Coronary artery disease | 4 (33) | 4 (36) |
| Diabetes | 5 (42) | 2(18) |
| Hypertension | 7 (58) | 6 (36) |
| Body mass index (BMI)* | 28.0 (25.7-30.4) | 29.8 (26.0-35.8) |
| Systolic BP, mmHg | 137 (112-151) | 142 (129-153) |
| Diastolic BP, mmHg | 69 (57-79) | 74 (68-91) |
| Heart rate, beats/min | 86 (69-93) | 84 (77-94) |
| Creatinine, umol/L | 127 (88-165) | 84 (73-109) |
| eGFR, mL/min/1.73 m^2^ | 43 (30-75) | 55 (44-74) |
| NT-pro BNP, ng/L* | 3116 (1139-5322) | 1736 (646-4420) |
| Left ventricular ejection fraction (LVEF)** | 55 (47-64) | NA |

**Table S3.** **Baseline characteristics of randomised patients.**  Values are median (IQR) or n (%). BP = blood pressure; NT-pro BNP = N terminal pro B-type natriuretic peptide; eGFR = estimated glomerular filtration rate; HF = Heart Failure;  Chronic pulmonary disorders= COPD, Interstitial lung disease, pleural plaques, bronchiectasis. BMI calculated from height and weight measurements (taken at same time, variable during course of admission); *12/12 and 10/11 for BMI and NTproBNP data completeness ** 7/12 and 3/11 for LVEF data completeness

| **Trial ID** | **Primary diagnosis**  **(Hospital episode statistics, ICD-10)** | **HF as primary or secondary diagnosis (OMOP)** | **HF as primary or secondary diagnosis (OMOP and EHR review)** |
| --- | --- | --- | --- |
| 7 | Congestive Heart Failure (I50.0) | 1 | 1 |
| 8 | Cellulitis of other parts of limb (L03.1) | 0 | 1 |
| 10 | Asthma, unspecified (J45.9) | 1 | 1 |
| 17 | HF, unspecified (I50.9) | 1 | 1 |
| 18 | Left ventricular failure (I50.1) | 1 | 1 |
| 24 | Chest pain, unspecified (R07.4) | 0 | 1 |
| 35 | Congestive Heart Failure (I50.0) | 1 | 1 |
| 40 | Congestive Heart Failure (I50.0) | 1 | 1 |
| 41 | Congestive Heart Failure (I50.0) | 1 | 1 |
| 55 | HF, unspecified (I50.9) | 1 | 1 |
| 65 | Acute renal failure, unspecified (N17.9) | 1 | 1 |
| 73 | Congestive Heart Failure (I50.0) | 1 | 1 |
| 74 | Left ventricular failure (I50.1) | 1 | 1 |
| 79 | Left ventricular failure (I50.1) | 1 | 1 |
| 94 | Atrial fibrillation and atrial flutter, unspecified (I48.9) | 1 | 1 |
| 95 | Left ventricular failure (I50.1) | 1 | 1 |
| 99 | Congestive Heart Failure (I50.0) | 1 | 1 |
| 100 | Congestive Heart Failure (I50.0) | 1 | 1 |
| 107 | Hyperglycaemia, unspecified (R73.9) | 1 | 1 |
| 111 | Essential (primary) hypertension (I10) | 0 | 1 |
| 115 | Sepsis due to Staphylococcus aureus (A41.0) | 1 | 1 |
| 122 | Malignant neoplasm: middle third of oesophagus (C15.4) | 0 | 0 |
| 126 | Congestive Heart Failure (I50.0) | 1 | 1 |

**Table S4.** **EHR derived diagnoses of randomised patients.** EHR = Electronic health record. ICD = International Classification of Disease. OMOP = Observational Medical Outcomes Partnership. The OMOP concepts used to define HF is provided as an additional supplemental codelist

| **Trial ID** | **Physician** | **Physician <48h** | **Nursing <48h** | **Nursing**  **<36h** | **Nursing**  **<24h** | **Nursing**  **<12h** | **Total**  **Adherence** |
| --- | --- | --- | --- | --- | --- | --- | --- |
| 8 | 1 | 1 | 0 | 0 | 0 | 0 | 2 |
| 10 | 1 | 1 | 1 | 1 | 1 | 1 | 6 |
| 18 | 1 | 1 | 1 | 1 | 1 | 1 | 6 |
| 24 | 0 | 0 | 0 | 0 | 0 | 0 | 0 |
| 41 | 0 | 0 | 0 | 0 | 0 | 0 | 0 |
| 55 | 1 | 1 | 1 | 1 | 0 | 0 | 4 |
| 79 | 1 | 0 | 0 | 0 | 0 | 0 | 1 |
| 95 | 1 | 1 | 1 | 1 | 1 | 1 | 6 |
| 100 | 1 | 1 | 1 | 1 | 1 | 1 | 6 |
| 99 | 1 | 1 | 1 | 0 | 0 | 0 | 3 |
| 94 | 1 | 1 | 1 | 0 | 0 | 0 | 3 |
| 7 | 1 | 1 | 0 | 0 | 0 | 0 | 2 |
| 35 | 1 | 0 | 0 | 0 | 0 | 0 | 1 |
| 73 | 1 | 1 | 1 | 1 | 1 | 1 | 6 |
| 74 | 1 | 1 | 0 | 0 | 0 | 0 | 2 |
| 107 | 1 | 1 | 1 | 1 | 1 | 0 | 5 |
| 111 | 1 | 1 | 0 | 0 | 0 | 0 | 2 |
| 115 | 1 | 1 | 1 | 1 | 0 | 0 | 4 |
| 122 | 1 | 0 | 0 | 0 | 0 | 0 | 1 |
| 126 | 1 | 1 | 0 | 0 | 0 | 0 | 2 |
| 17 | 1 | 1 | 0 | 0 | 0 | 0 | 2 |
| 65 | 1 | 1 | 1 | 1 | 0 | 0 | 4 |
| 40 | 1 | 1 | 1 | 0 | 0 | 0 | 3 |

**Table S5: Degree of EHR evidence of treatment implementation.** Clinician acknowledgment (Physician or Nursing) was assessed and scored according to the time from decision to admit.

| **Trial ID** | **Allocation** | **Adherence** | **Oral fluid intake (ml)** | **Data entries for oral intake** |
| --- | --- | --- | --- | --- |
| 8 | No Restrict | 1 | 50 | 1 |
| 10 | No Restrict | 1 | 1020 | 6 |
| 18 | No Restrict | 1 | 650 | 4 |
| 24 | No Restrict | 0 | 100 | 1 |
| 41 | No Restrict | 0 | 1185 | 7 |
| 55 | No Restrict | 1 | 1975 | 14 |
| 79 | No Restrict | 1 | 955 | 8 |
| 95 | No Restrict | 1 | 370 | 3 |
| 100 | No Restrict | 1 | 2000 | 10 |
| 99 | No Restrict | 1 | 0 | 0 |
| 94 | No Restrict | 1 | 0 | 0 |
| 7 | Restrict | 1 | 1085 | 7 |
| 35 | Restrict | 1 | 1050 | 9 |
| 73 | Restrict | 1 | 1720 | 12 |
| 74 | Restrict | 1 | 1680 | 13 |
| 107 | Restrict | 1 | 1050 | 5 |
| 111 | Restrict | 1 | 250 | 1 |
| 115 | Restrict | 1 | 580 | 5 |
| 122 | Restrict | 1 | 1350 | 8 |
| 126 | Restrict | 1 | 1600 | 7 |
| 17 | Restrict | 1 | 1250 | 3 |
| 65 | Restrict | 1 | 1699 | 9 |
| 40 | Restrict | 1 | 0 | 0 |

**Table S6.** **Raw data for Primary outcome: recruitment and treatment effect difference**

Adherence = physician acknowledgement of allocation alert

| **Trial ID** | **Treatment allocation** | **Length of stay (d)** | **Total Documented fluid intake (ml)** | **Total Documented output (ml)** | **Fluid balance (ml)** | **Weight change 48h after allocation (g)** |
| --- | --- | --- | --- | --- | --- | --- |
| 8 | No Restrict | 2 | 50 | 0 | +50 | NA |
| 10 | No Restrict | 6 | 1020 | 350 | +670 | -3596 |
| 18 | No Restrict | 1 | 650 | 1688 | -1038 | NA |
| 24 | No Restrict | 8 | 650 | 1140 | -490 | NA |
| 41 | No Restrict | 2 | 1185 | 0 | +1185 | NA |
| 55 | No Restrict | 6 | 4245 | 9995 | -5750 | +567 |
| 79 | No Restrict | 13 | 4245 | 14980 | -10735 | NA |
| 94 | No Restrict | 3 | 750 | 3212 | -2462 | NA |
| 95 | No Restrict | 5 | 370 | 5685 | -5315 | -2243 |
| 99 | No Restrict | 5 | 120 | 1550 | -1430 | NA |
| 100 | No Restrict | 4 | 3600 | 10343 | -6743 | -7099 |
| 7 | Restrict | 8 | 5365 | 14896 | -9531 | +4753 |
| 17 | Restrict | 20 | 7100 | 26085 | -18985 | NA |
| 35 | Restrict | 6 | 3900 | 1770 | +2130 | NA |
| 40 | Restrict | 9 | 900 | 4194 | -3294 | NA |
| 65 | Restrict | 9 | 7399 | 0 | +7399 | -612 |
| 73 | Restrict | 6 | 4270 | 8196 | -3926 | -1380 |
| 74 | Restrict | 13 | 6580 | 14775 | -8195 | -1870 |
| 107 | Restrict | 17 | 16530 | 6601 | +9929 | +462 |
| 111 | Restrict | 2 | 250 | 0 | +250 | -1089 |
| 115 | Restrict | 22 | 8920 | 8593 | +327 | NA |
| 122 | Restrict | 8 | 3850 | 6525 | -2675 | NA |
| 126 | Restrict | 9 | 4710 | 0 | +4710 | -7557 |

**Table S7. Total oral fluid intake across admission, output, balance and weight change.
NA = Not applicable. For**

| **Trial ID** | **Treatment allocation** | **Prior Furosemide Dose (mg)** | **Furosemide/ Furosemide Equivalent Day 1 dose** | **Furosemide/ Furosemide Equivalent Day 2 dose** | **Baseline Cr (micromol/L)** | **Change in Cr Day 1**  **(micromol/L)** | **Change in Cr Day 2**  **(micromol/L)** |
| --- | --- | --- | --- | --- | --- | --- | --- |
| 8 | No Restrict | 80 | 20 | NA | 74 | -9 | NA |
| 10 | No Restrict | 40 | 120 | 120 | 64 | NA | NA |
| 18 | No Restrict | 40 | NA | NA | 86 | NA | NA |
| 24 | No Restrict | 40 | 40 | 40 | 248 | -122 | -6 |
| 41 | No Restrict | 80 | 40 | NA | 82 | NA | NA |
| 55 | No Restrict | 60 | 120 | 80 | 224 | +1 | +8 |
| 79 | No Restrict | 280 | 20 | 20 | 75 | NA | NA |
| 94 | No Restrict | 80 | 80 | NA | 105 | NA | NA |
| 95 | No Restrict | 40 | 120 | 80 | 79 | -5 | +7 |
| 99 | No Restrict | 120 | 120 | NA | 100 | NA | NA |
| 100 | No Restrict | 80 | 80 | 80 | 87 | +10 | NA |
| 7 | Restrict | 200 | 120 | 120 | 92 | +5 | +4 |
| 17 | Restrict | 40 | 80 | 120 | 215 | -143 | NA |
| 35 | Restrict | 40 | 80 | 200 | 363 | +49 | -203 |
| 40 | Restrict | 40 | 40 | 40 | 135 | +2 | -11 |
| 65 | Restrict | 240 | 240 | 240 | 171 | NA | +13 |
| 73 | Restrict | 40 | 160 | 80 | 80 | +9 | -11 |
| 74 | Restrict | 40 | 40 | 40 | 105 | -5 | +32 |
| 107 | Restrict | 80 | 40 | 80 | 468 | -188 | -26 |
| 111 | Restrict | 80 | 40 | NA | 98 | NA | NA |
| 115 | Restrict | 280 | 240 | NA | 127 | 0 | NA |
| 122 | Restrict | 40 | 40 | NA | 139 | NA | NA |
| 126 | Restrict | 120 | 120 | 160 | 197 | NA | NA |

**Table S8.** **Diuretic dose and change in renal function.** NA= not applicable. For furosemide dosing data, this was as a result of lack of prescription rather than mi

## Supplementary Figures


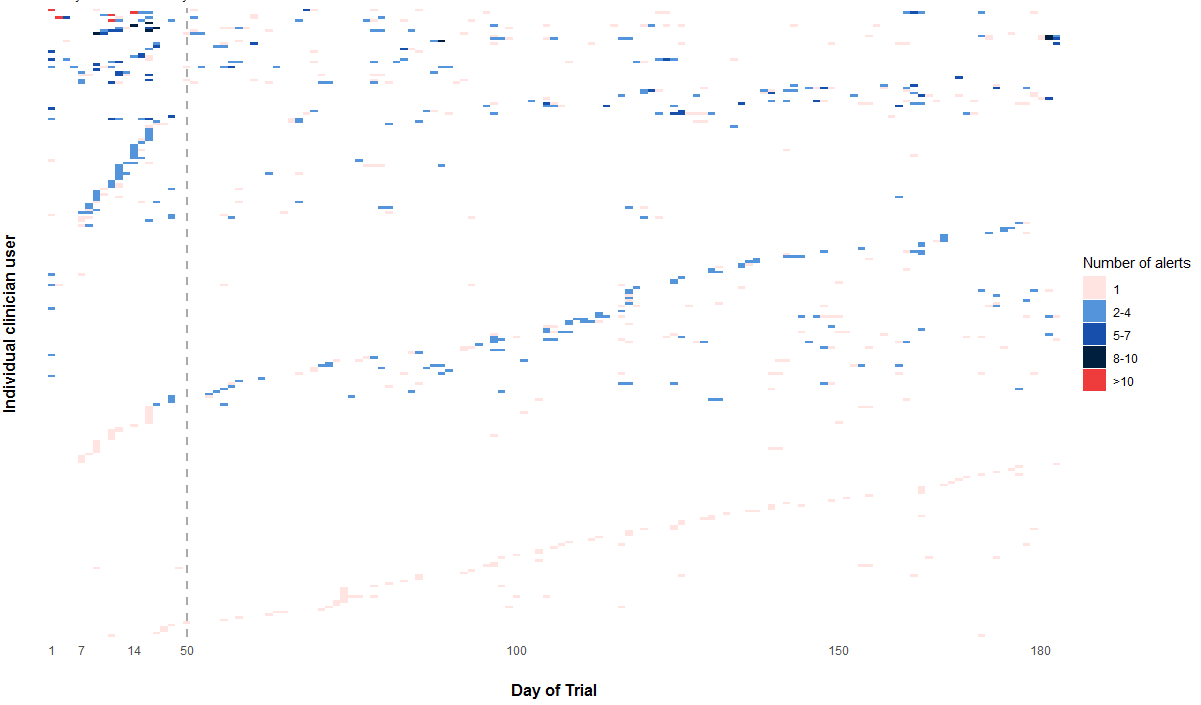


**Figure S1 Burden of THIRST alert received by users before and after protocol amendment.** Each row represents a unique staff user. Between Day 1 and Day 20 of study recruitment, the alert was triggered on ‘Open sidebar’ and ‘Open chart’. Between day 21-49, the alert was switched to shadow mode to allow modification to the trigger condition. Between day 50-183, only ‘Open Sidebar’ triggered the alert.

**
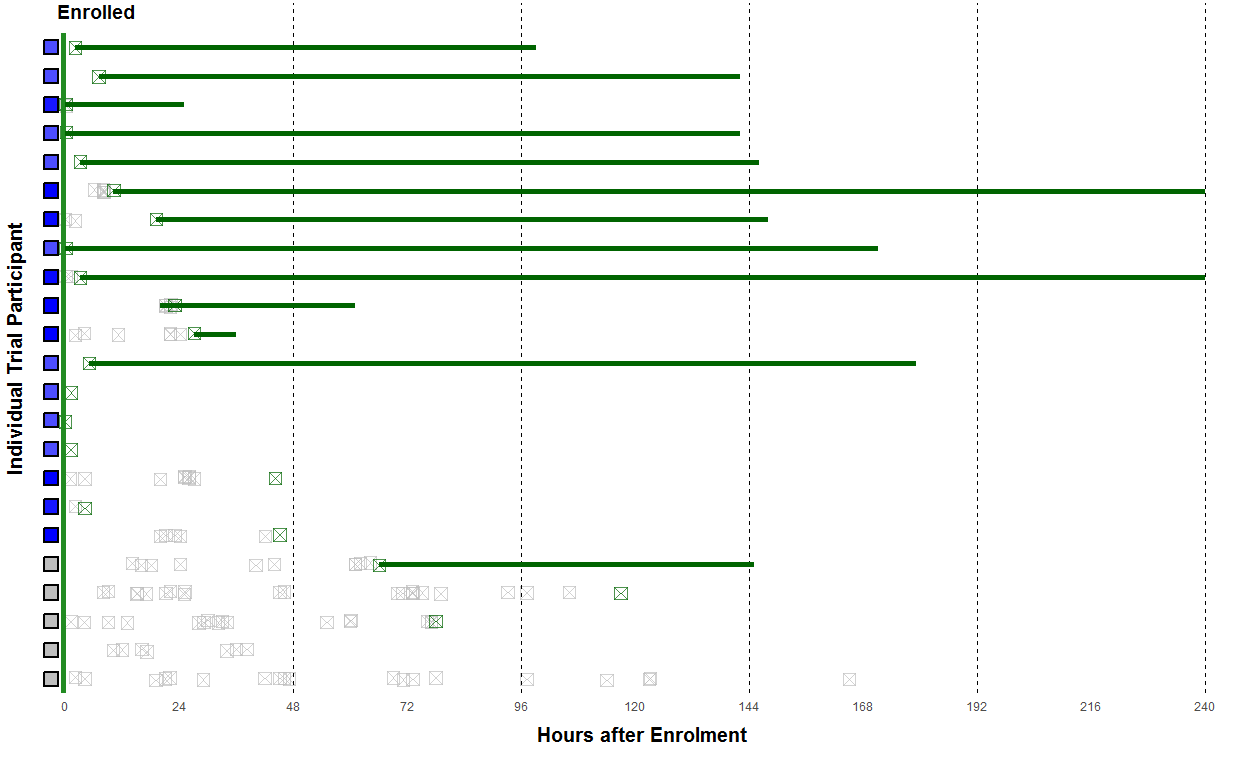
**

**
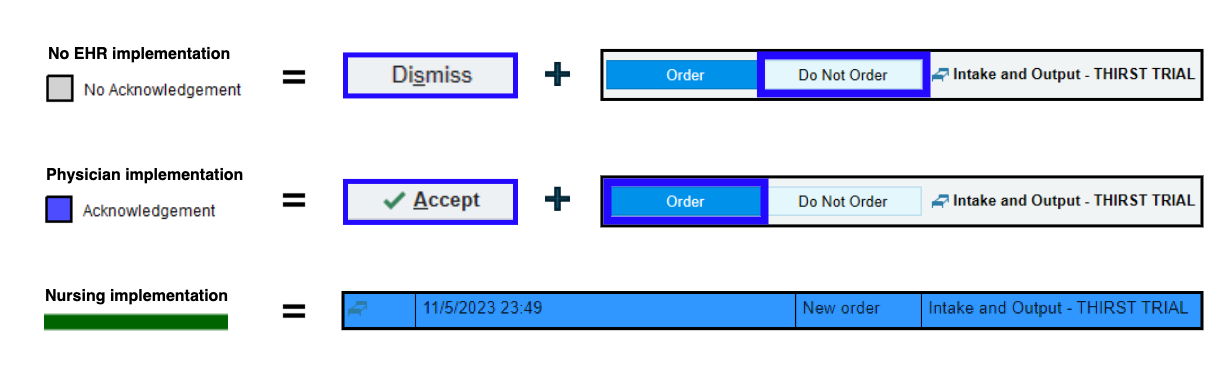
**

**Figure S2**. **Documented evidence of adherence to allocation after enrolment.** For each participant, enrolment is represented at hour 0 with the vertical green line. Grey square with cross = physician dismissal of allocation alert. Green square with cross = physician acknowledgement of allocation alert. © 2024 Epic Systems Corporation

**
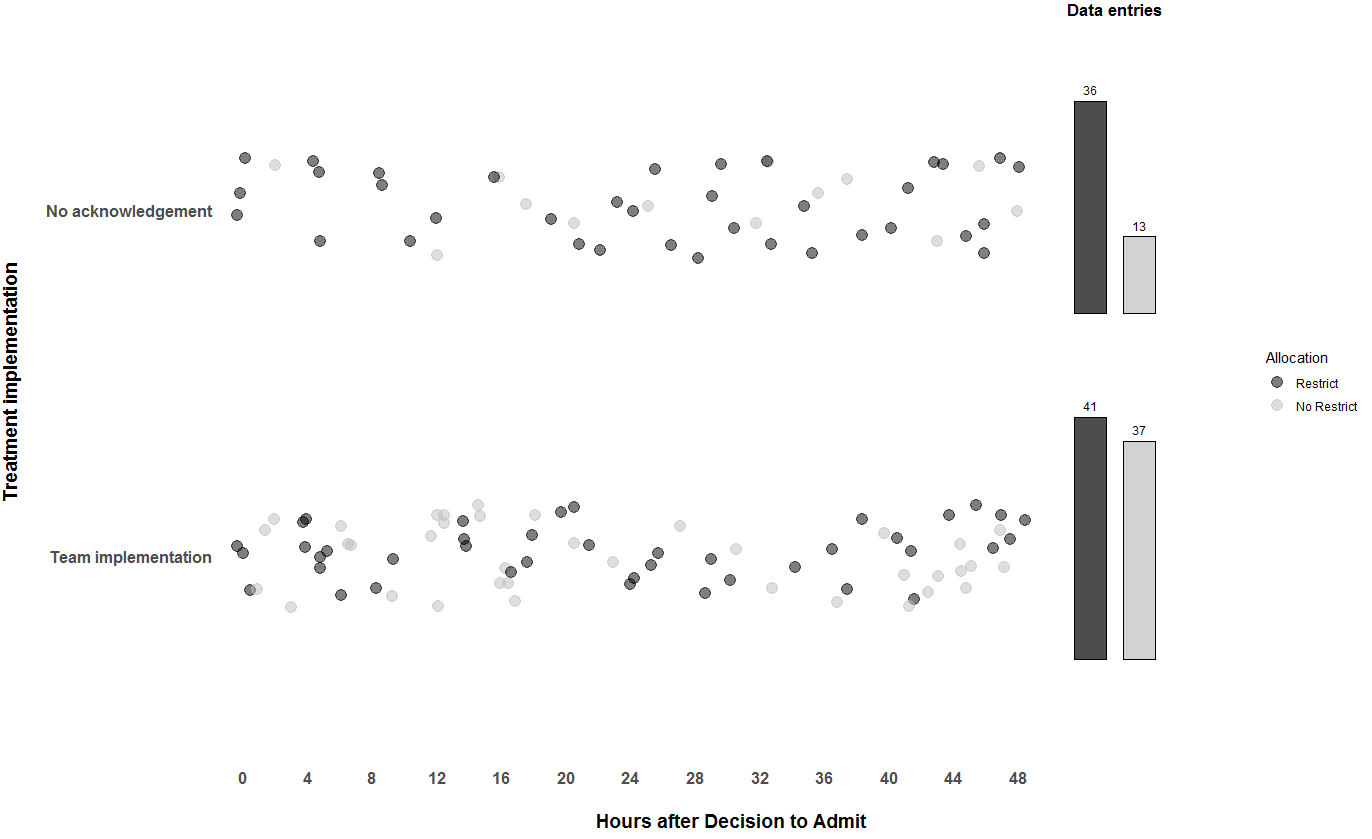
**

**Figure S3. Documented entries of oral intake and overall intake volume.** Number of EHR data entries of oral intake by presence or absence of documented implementation of treatment allocation.

**
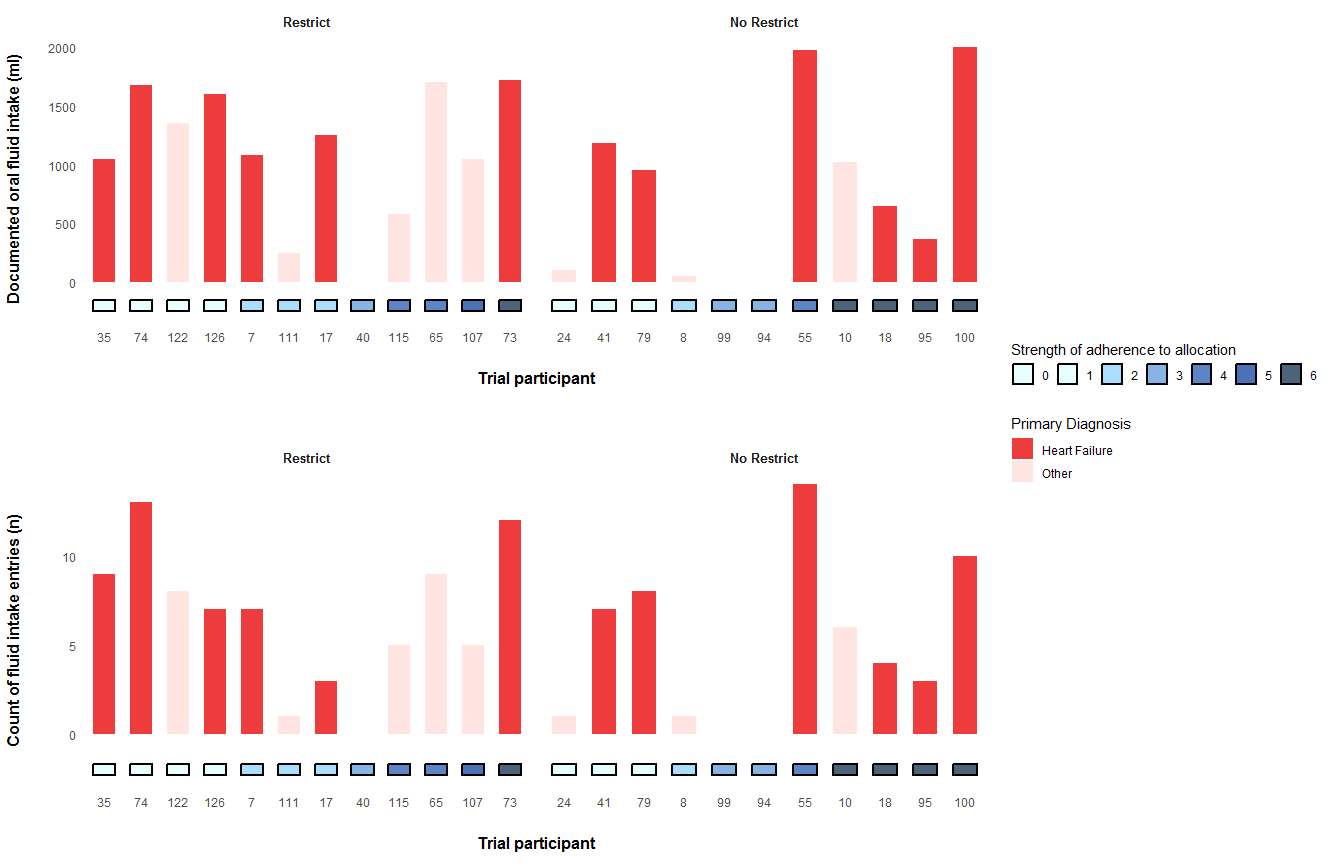
**

**Figure S4.** Documented oral intake and data entries according to adherence to treatment allocation and heart failure status

## Supplementary methods

### Protocol amendment

A non-substantial amendment (Sponsor amendment reference: NSA01) was granted to extend the study recruitment period by three months. This was required to debug some issues related to the alert triggering conditions. Initial observations made by the trial management group included a concern for alert fatigue. The trigger rule of ‘Open order sidebar’ AND ‘Open Patient Chart’ was assessed as being too sensitive, with the latter condition causing repeated presentations of the alerts to users, who were likely opening the patient chart for other reasons. For 24 eligible admissions during the first recruitment period (03/05/23-22/05/23), 404 alerts were triggered (median 12 alerts per admission (IQR 6-22)). A decision was taken to move the alerts to ‘not shown to users’ at the study site while the necessary change was made to remove the ‘Open Patient Chart’ condition. In the second recruitment period (21/06/23-01/11/23), there were 774 enrolment alerts triggered for 117 eligible admissions, median 6 alerts per admission (IQR 2-9)). In addition, between the first and second recruitment periods, we corrected a bug noted in the first recruitment window where after a user clicked ‘no’, the alert was still presented – this did not affect the number of patients enrolled into the trial (affected participants ID27, 29, 32, 45, 56 did not have any different subsequent actions. In total, there were 153 days (~5 months) of patient recruitment.

Eligible patient population

There were 145 eligible admissions during the trial recruitment window. There were an additional ten patient admissions where no trial enrolment occurred but baseline data was ineligible for use due to patient registration in the NHS data opt-out scheme.

### Trial covariates, concept definitions and HF diagnosis

A separate datasheet is included that contains all data items extracted from the EHR system for the THIRST trial, in the Observational Medical Outcomes Partnership (OMOP) Common Data Model (CDM) format. For applicable items, the relevant International Classification of Disease or SNOMED CT terms are included.

For echocardiographic left ventricular ejection fraction data, this was based on a separate raw EHR data extract. Echocardiographic data within 12 months of eligible index admissions were included and where multiple echocardiograms existed, the most recent data were used. Left ventricular ejection fraction (LVEF) was determined from numeric structured data fields rather than free text or report summaries – see “THIRST_codelist supplement” for the list of data fields used.

For the four patients who did not have a coded diagnosis of Heart failure, manual case note review and diagnosis adjudication was conducted by two authors (YC and RTL) to identify whether there was documentation of heart failure or substitute phrases in the free text, or whether structural abnormalities or HF manifestations were noted in echocardiography reports in conjunction with elevated NT-proBNP to satisfy HF diagnostic criteria.
